# Indents

**Published:** 1912-05-15

**Authors:** 


### INDENTS.

**☞Brief Articles of Technical or Scientific Value will receive notice and comment. Contributions invited.**

*Fees.* Without attempting to elaborate upon the sociological, economic or philosophical questions that may be specifically involved, few will dispute that, generally speaking, the fees of a dentist should be large enough to, with a fair practice, pay the necessary costs of conducting his business and leave a sufficient balance for his wages to provide necessities for himself and family, at least.

These necessities, it will probably be granted, mean sufficient food, clothes and shelter and enough "schooling" for his children, if he has any, to give them a fair start in life.

Leisure, recreation and rest within reasonable limits, might well be classed as necessities also, but seem to be rather more conditional in their nature than the fundamental necessary ones first enumerated.

Broadly speaking, if it is granted that the necessities of a dentist should determine the size of fees, the more dentists there were and the smaller the practice, the higher would the individual fees have to be, while the poorer equipped the office, the smaller the family of the dentist and the cheaper the rent, the less they would be.—*Digest.*

*Separating Compound Impressions.* Heat the impression and model in hot water and then remove and allow cold water to run onto the compound two or three seconds. The model can then be easily removed and none of the compound will adhere to the model.—*N. J. Dental Journal.*

*Crowns: Bridges.* The objectionably wide cement line found under practically every all-porcelain crown may be reduced to the same infinitesimal minimum as is present around a well-fitted gold inlay by interposing a disk-like casting of gold on the dowel between the crown and the root; the wax pattern for this disk having been formed by pressure between the cut ends of the crown and root. We may also by this means preserve nature's continuity of surface at the neck of the tooth, and at the same time avoid causing the intense pain incident to scaling the stump and fitting a band. After three and a half years of general use of this type of mount for the all-porcelain crowns, we have yet to be shown wherein the technique is defective.—J. G. Lane, D.D.S., *The Dental Brief.*

*New Metal.* Andrew Gordon French, a metallurgist, who earlier in the year discovered the presence of platinum minerals in the Kootenal ores, in Alaska, has announced the discovery of a metal hitherto unknown to science. He has named it canadism.

The metal is found in large quantities and is of high commercial value. It belongs to the platinum group and it is said will be largely employed in scientific work.

With platinum at $46.00 per ounce here is a chance for manufacturers of artificial teeth to try this new metal.—*Scrap Book.*

*Some Walker.* Alfred Owre, dean of the dental department of the University of Minnesota, walked a distance of 4,125 miles during 1911, according to a pedometer which he constantly carried with him. Dr. Owre has the distinction of having never boarded a street car, with but few exceptions, to and from the university, during the past eleven years. Dean Owre started his pedometer on its record for 1912 by walking a distance of 19 miles.

*Foreign Students.* Many foreign countries are represented by students at the University of Pennsylvania, which for the last five or six years has had the largest number of foreign students of any educational institution in the United States.

This year there are about 240 foreign students, from forty-three different countries, every continent being represented many times. Half of the total number of foreigners are studying dentistry. Graduates of the Pennsylvania dental school are in strong demand abroad.

*The blood Pressure in Anesthesia.* It may be said that the effects of an anesthetic on the circulation are of little importance unless the blood pressure is altered, and that any disturbance of the blood pressure caused by an anesthetic is an effect of much moment, well worthy of careful study. An anesthetic which affects the blood pressure but slightly is, other factors being equal, to be preferred to one which affects it more. The authors have joined their efforts in the present research, with the following objects in view: (1) To observe and compare the effect of the administration of various anesthetic agents on systolic pressure; (2) to note any variations in the effect produced by different methods of administration, and (3) to ascertain the conditions favorable to the production of the minimal disturbance of systolic pressure during the .induction of anesthesia. The following agents were employed: Chloroform; ether; nitrous oxid by re-breathing, with valves, or preceded by oxygen; nitrous oxid and ether in sequence; oxygen, gas, and ethyl chlorid in sequence. The operations, save those done with chloroform and ether, were dental, and the patients were seated. The carefully tabulated results of each method employed led to the following conclusions: That there is no practical difference between the available anesthesia brought about by nitrous oxid when re-breathing is allowed and that available when nitrous oxid is given with valves, but that the effects of asphyxia are much more marked in the latter method. The asphyxial element present in the re-breathing method can be avoided by the previous inhalation of a gallon of oxygen, but the period of available anesthesia is often shortened a few seconds. Ethyl chlorid causes marked disturbances of the circulation, and in large doses may bring about a dangerous fall of systolic blood pressure by inhibition of the heart. This fall is prevented by the previous inhalation of gas, the effect then being a constant rise, probably, in part at least, due to asphyxia. The slight asphyxial element in gas and ethyl chlorid anesthesia can be eliminated by the previous administration of a gallon of oxygen without any shortening of the period of available anesthesia.—*Cosmos from British Journal of Dental Science.*

*Making Perfectly Fitting Clasps.* Ill-fitting clasps are the cause of many troubles in sound teeth as we all know; therefore a perfectly fitting clasp is a most important factor in the making of a satisfactory denture. The first essential is a perfect model of the tooth to be banded, and this should be obtained from a plaster impression. A piece of thin tin foil is burnished around the tooth, and on it a piece of thin wax plate is pressed, and trimmed up with a sharp knife to the desired breadth and shape; the band and foil are removed together from the tooth, the foil is peeled off, and the piece invested and cast in the usual way, using band gold. In this way a perfectly fitting clasp is obtained, which conforms to the contour of the tooth.—P. S. Humm, *Brit. Jour. of Dental Science.*

*Cast Gold Inlays.* In using occlusal gold inlays, the operator will find that a piece of rubber dam just large enough to cover the wax model, placed between the occluding teeth while the wax is soft, will make the articulating surface of the inlay occlude exactly, and there will be no grinding to do when the inlay is finished and polished.—*Dental Cosmos.*

*Bacteria of the Mouth and the Value of Tooth-brushes and Tooth-Pastes.* On waking in the morning the mouth has been found to contain about 3000 million bacteria capable of being removed by a fivefold rinsing with 25 ccm. of water each time. After ordinary washing of the teeth with a hard tooth-brush, about one-fourth the number could still be rinsed away. After using, on another occasion, a tooth-paste containing one of the germicidal essential oils which have a very high phenol coefficient, 120 million only were left.—E. C. Bousfield, *Lancet.*

*A Laboratory Help.* A piece of linoleum five or six inches square will be found to be a very convenient help in pouring up plaster models. It gives a flat base to the model, can be easily "peeled" off when the plaster is set, and does not chip or break as does a glass slab. It is a proper companion to the rubber bowl.—C. M. Torrence, Frankfurt, Germany.

*Care of Tooth Brush.* The tooth brush may be kept sterile by taking a large size test tube and constricting it about one inch from the bottom, providing it with a rubber cork as a stopper and placing a little formalin tablet or formaldehyde, in the lower chamber. It will then become sterile without injury to the brush.

*A Suggestion About Base Plates.* When the base plate is returned to the cast, and it is found that the palatine portion of the base plate is not in contact with the cast, the operator often attempts a readoption of this part of the plate by pressing it down, and in so doing draws the impression of the occluding nearer the centre of the mouth. When the denture is finished the articulation of the artificial teeth with the natural is quite different from their arrangement on the articulator.

No matter how much time you may spend in taking bites or articulating the teeth, there always follows a slight change in the articulation.—C. B. Voigt.

*Cotton.* When loading your cotton holder, cut the end of the cotton as it comes from the roll crosswise into strips ½ to ¾ inches wide. Pack these into the holder and when using small bits of cotton, you will be pleased to see how nicely you can get just what you want, no more.—C. E. Allen, D.D.S., Chicago, Ills.

*Removing Modeling Compound from Trays.* Paint the modeling compound and tray with olive oil; then hold it over the Bunsen flame and while it is still hot wipe the compound from the tray with a cloth. —*N. J. Dental Journal.*

